# Modelling the Impact of Atherosclerosis on Drug Release and Distribution from Coronary Stents

**DOI:** 10.1007/s10439-015-1456-7

**Published:** 2015-09-18

**Authors:** C. M. McKittrick, S. Kennedy, K. G. Oldroyd, S. McGinty, C. McCormick

**Affiliations:** Department of Biomedical Engineering, University of Strathclyde, Glasgow, UK; Institute of Cardiovascular and Medical Sciences, University of Glasgow, Glasgow, UK; West of Scotland Region Heart and Lung Centre, Golden Jubilee National Hospital, Dunbartonshire, UK; Department of Mathematics and Statistics, University of Strathclyde, Glasgow, UK

**Keywords:** Drug-eluting stents, Atherosclerosis, Drug uptake, Drug distribution, *In vitro* models, *In vivo* models, Mathematical models, Computational models, Drug transport, Arteries

## Abstract

Although drug-eluting stents (DES) are now widely used for the treatment of coronary heart disease, there remains considerable scope for the development of enhanced designs which address some of the limitations of existing devices. The drug release profile is a key element governing the overall performance of DES. The use of *in vitro*, *in vivo*, *ex vivo*, *in silico* and mathematical models has enhanced understanding of the factors which govern drug uptake and distribution from DES. Such work has identified the physical phenomena determining the transport of drug from the stent and through tissue, and has highlighted the importance of stent coatings and drug physical properties to this process. However, there is limited information regarding the precise role that the atherosclerotic lesion has in determining the uptake and distribution of drug. In this review, we start by discussing the various models that have been used in this research area, highlighting the different types of information they can provide. We then go on to describe more recent methods that incorporate the impact of atherosclerotic lesions.

## Introduction

Although drug-eluting stents (DES) have revolutionised the treatment of coronary heart disease, their performance is severely limited in some patient groups,^3^ and lesion types.^2^ Moreover, delayed endothelialisation remains a concern.^24^ Consequently, considerable research remains dedicated towards the development of improved DES. There are a number of key aspects of DES that contribute to the overall performance of the device, and each must be optimised, in an integrated fashion, for successful performance. The stent platform, material and coating clearly play an important role in performance,^34^^,^^52^^,^^54^ with recent improvements including the use of thinner struts and a variety of different metal alloys.^19^^,^^39^ Most recently, fully bioresorbable stents have been developed, consisting entirely of biodegradable polymers or metals [see Wiebe *et al*. (2014) for a recent review].^72^ The drug that is delivered from the stent is also of crucial importance, and although a great variety of drugs have been investigated [see Khan *et al*. (2012) for a recent review],^29^current clinically used DES rely on the use of sirolimus analogues and paclitaxel. In this review, we focus on the challenge of optimising the release and uptake of drug from the stent surface into the vessel wall. We will firstly describe the current models used to characterise this aspect of DES design and performance, before going on to describe recent efforts to incorporate atherosclerosis into such models.

## Modelling Drug Release and Distribution

**Figure 1:**
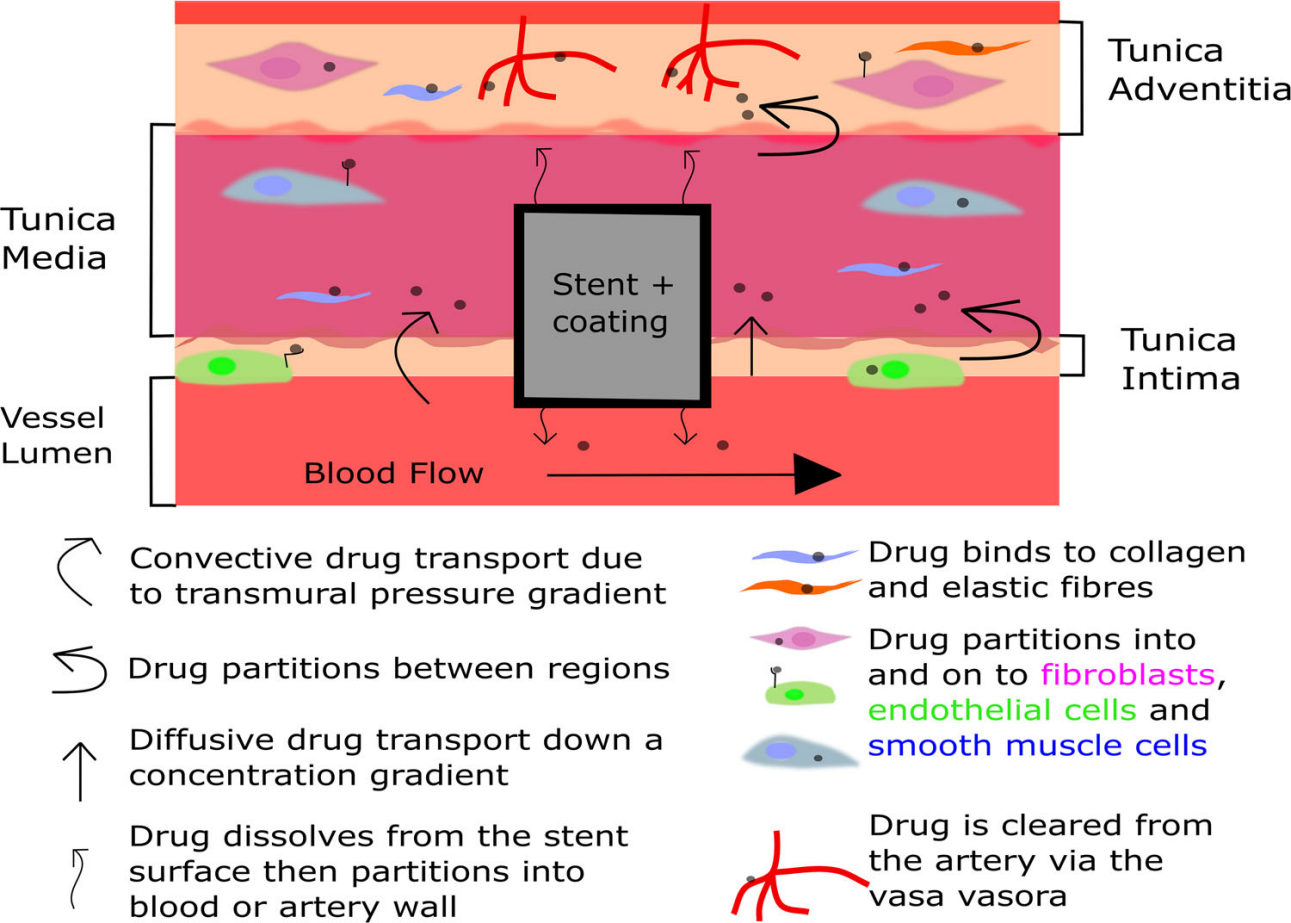
A simplified diagram illustrating the key arterial ultrastructures and the transport forces involved in drug distribution within a stented artery. Drug is transported through the vessel wall by various forces and partitions to both specific and non-specific binding sites. Figure adapted from Yang* et al*. 2006.^73^

Stent-based drug release and the subsequent uptake and distribution within arterial tissue is a complex phenomenon, involving a series of different but often interdependent mechanisms (Fig. 1). Several models are available, which attempt to describe at least some aspects of this phenomenon. Schwartz *et al*. 2008^56^ made a series of recommendations on a variety of pre-clinical tests that should be performed during DES development and evaluation. These range from simple *in vitro* dissolution tests through to whole animal studies. In the first part of this review, we will describe the current state of the art within each test type. We will also consider the emerging role that *ex vivo* models and computational techniques are playing in this area.

### *In Vitro* Models

*In vitro* drug release profile models are an integral part of the DES development process. They are particularly useful for establishing the reproducibility of the coating procedure and drug release. This can be readily assessed by using accelerated release methods, where solvents and/or surfactants are added to the release medium to increase the rate of drug release.^28^ In addition to their importance for quality control purposes, such methods can also provide useful insights into the mechanisms governing drug release from the coating. Mechanistic information can also be gained from the use of *in vitro* models that seek to mimic *in vivo* drug release. A number of requirements need to be met in order that *in vitro* release resembles the *in vivo* situation as closely as possible and these should take into consideration the drug being investigated (see Schwartz *et al*. 2008 and Schwartz *et al*. 2002 for recommendations).^55^^,^^56^

The simplest *in vitro* drug release methods incubate the stent, or stent substitute, in release medium and transfer the sample to fresh medium at predetermined time points. It is generally recommended that the volume of the release medium should be selected to ensure infinite sink conditions are maintained as closely as possible, thus better mimicking the *in vivo* situation and preventing the drug from saturating the release medium, which would affect the dissolution rate.^55^^,^^56^ The release media used can alter the release profile, as the drug and/or polymer used can be sensitive to changes in media composition, with such effects being a common feature of accelerated drug release assays. As an example, the addition of solvents to the release media increased solubility and dramatically altered *in vitro* paclitaxel release from the TAXUS stent, effects that were dependent on both the solvent type and concentration used.^27^ A similar effect was highlighted by Kamberi *et al*. 2009, who found that increasing acetonitrile concentration within the release medium increased the total drug elution from an everolimus-eluting stent.^28^ Furthermore, the porosity of the polymer coating can be affected by the presence of solvent in the release media with possible implications on the drug release profile. It was found that the porosity of the polymer coating was increased in stents placed in dissolution media containing acetonitrile, whilst the structure remained unaltered in control media.^28^

In the case of sirolimus, the drug release profile has been shown to be dependent on both drug stability and solubility in the release medium. Indeed, cumulative release of sirolimus was maximal in a normal saline-isopropyl alcohol mix, compared to both phosphate buffer (PB) and phosphate buffered saline (PBS) over an extended 75 day period from a polymer free stent. This difference was most likely due to the pH of PB and PBS (being pH 7.4), causing hydrolysis of the sirolimus.^30^^,^^45^

Just as careful selection of the release media can provide accelerated release; it can also be used to produce *in vitro* release profiles that are useful predictors of *in vivo* release. This is particularly important when the drug being investigated is known to bind to proteins, and/or undergoes degradation. In human plasma, cumulative release of sirolimus reached a lag phase by 1 week and only increased marginally up to the 25 day end point of the experiment, a pattern thought to be due to degradation of the drug.^30^ Long term release of everolimus into porcine serum was assessed in order to produce a biorelevant release profile.^28^ To circumvent the problem of detection and degradation of the drug in serum the investigators inferred the serum concentration at each sampling point from the drug remaining on the stent. Additionally, this release profile correlated well with a short-term release profile which used solvents in the release medium. Such an approach allows for a reduction in the time required to obtain robust release profiles from weeks to hours.

The standard *in vitro* dissolution tests we have described so far lack the presence of a stent-artery interface. Whilst these can provide useful information on the reproducibility of the coating process and indeed on the mechanisms of drug release,^35^ the release profile can only be indicative of the likely *in vivo* situation and cannot account for transfer of drug from the stent surface into tissues. More recent developments have attempted to address this limitation through the use of flow-cells that mimic the stent-artery interface.^46^^,^^57^ These models contain a chambered hydrogel with a hollow central aperture, into which a stent can be deployed. This allows quantification of drug diffusion and distribution into the hydrogel compartment over time. A closed loop allows continuous flow through of media which can be serially sampled and drug release into the compartment measured.^46^ Additional work with this model has described spatial distribution of model substances within the hydrogel compartment which agreed well with finite element analysis of diffusion coefficients.^57^

An alternative model has been developed to assess the impact of flow on drug release from a single stent strut through a hydrogel.^47^ The primary purpose of this work was to validate the findings from computational modelling that highlighted the contribution of flow around stent struts to drug uptake and distribution. Furthermore, this model sought to overcome the limited ability of computational models to incorporate the complex dynamics of pulsatile flow, and highlighted the importance of strut geometry on drug distribution. Distribution was dependent on the strut aspect ratio which determined the magnitude of regions of flow recirculation, and therefore concentration of drug within these regions. Further work with this model highlighted the importance of flow rate, as halving the mean vessel Reynolds number increased the uptake of drug by around 30%. Additionally, computational modelling indicated that pulsatile flow had a negligible impact on the overall mass of drug uptake in an idealised stent placement. However, increasing degrees of strut malapposition led to a decrease in drug uptake and distribution due to increased magnitude of regions of flow recirculation, causing decreased drug concentration in these regions.^47^

The use of artery mimics in these models means they do not adequately capture the effects of specific drug binding sites, or physiological drug transport mechanisms. Furthermore, they do not replicate the multi-layered arterial wall structure and tend to neglect both anisotropic diffusion and the effects of transmural pressure gradient.^46^^–^48^,^^57^ However, there are model systems available in which some of these parameters can be independently assessed. For example, drug binding to specific sites can be readily evaluated *in vitro*. Early work by Khu *et al*. (2000)^31^ described the complex binding of paclitaxel using complimentary *in vitro* and computational modelling. They found both extracellular and intracellular binding to be dependent on drug loading concentration and cell density in a time dependent manner. Similarly, sirolimus has also been investigated *in vitro* and shown to bind to the intracellular target FK-binding protein 12 (FKBP-12).^4^ The cellular uptake and release of sirolimus have also been investigated, by incubating human venous smooth muscle cells in different sirolimus concentrations (5, 15 or 25 *µ*g/mL).^77^ Equilibrium was rapidly reached within 30 min of incubation, and the drug remained within the cells over a 5 h period, suggesting that binding had taken place although this was not measured directly. Drug release from the cells was rapid, with an equilibrium level achieved within 60 min and this was maintained for the remainder of the 5 h incubation period. The data from these experiments was fitted to a first order kinetics equation which indicated that drug transport into and out of the cells was predominantly governed by diffusion.

There is emerging research investigating the release of drug from stents in real time which has been made possible by the use of advanced imaging techniques. Using correlated confocal Raman and atomic force microscopy, Biggs *et al*.^5^ mapped the chemical composition and topography of the surface and subsurface of the CYPHER stent. They were able to correlate sirolimus elution with structural changes to the surfaces, where the formation of porous regions had taken place.^5^ Carlyle *et al*.^7^ employed a vessel simulating loop which incorporated flow analogous to a coronary artery, mimicking pressure, shear stress and velocity, and a biorelevant release medium, to investigate release from an absorbable sirolimus eluting stent coating (AC-SES). Here they imaged the stent, macroscopically, through the surface of the flow loop and visualised changes to the stent polymer coating which softened and dispersed over time. Computational modelling indicated that polymer dispersion would lead to greater lateral distribution of drug within the artery wall due to increased contact between the stent coating and the artery.^7^

### *In Vivo* Models

Ultimately, an improved understanding of DES drug release kinetics and mechanisms requires the use of animal models. Many such models have been used in the development and assessment of DES. These range from smaller species, including the mouse aorta,^1^^,^^11^ up to larger models such as the pig coronary artery.^69^^,^^70^ For pre-clinical assessment of safety, it is generally accepted that the pig coronary artery is the most appropriate model.^56^ It is beyond the scope of this review to consider each model in detail and our discussion will focus initially on the use of rabbit and pig stent models for investigation of drug pharmacokinetics. However, the use of smaller models may permit the investigation of specific aspects of disease, such as specific lesion subtypes and changes to the vessel ultrastructure, which may impact device performance.

#### Pig Models

The pig coronary artery model is recognised as the gold standard animal model.^56^ It is therefore widely used for pre-clinical safety and efficacy assessment although it has been less well used for characterising drug pharmacokinetics. Watt *et al*. 2013 characterised drug release from a novel polymer-free anti-oxidant, succinobucol-eluting stent. Drug remaining on the stent and drug within arterial tissue were measured at multiple time points up to 28 days. The elution of the drug *in vivo* displayed a biphasic release profile with 60% of load eluted within the first week with a further 20% over the next 2 weeks. This study also found that tissue succinobucol content reduced over time indicating that clearance rate exceeded elution rate.^70^ Carlyle *et al*.^7^ were able to demonstrate extended and controlled drug release from an AC-SES using the pig model. This study highlighted the value of assessment of drug release over long periods of time, since it was found that although 97% of the release had occurred by 45 days, drug levels were still found in significant quantities within arteries after 90 days.^7^

Tzafriri *et al*. 2012^64^ also used the pig model, alongside computational modelling, to investigate the pharmacokinetics of two different sirolimus-eluting stents. The study investigated the tissue drug binding eluted from the CYPHER and NEVO stents. Both devices eluted a similar amount of drug over a 30 day period *in vitro*, with the former displaying a biphasic release profile and the latter a linear steady elution rate. Despite this initial burst the authors found that CYPHER stents produced consistently lower sirolimus tissue content from 3 to 30 days. Computational modelling was used to address this apparent discrepancy and it was predicted that receptor saturation was similar in arteries implanted with either stent, which may help explain the similar clinical efficacy reported for both devices. The insights provided by this study, achieved through the combined use of *in vivo* and computational models, demonstrate the value of adopting a variety of complementary approaches to furthering our understanding of stent-based drug delivery.

#### Rabbit Models

The rabbit is a species routinely used for the investigation of atherosclerosis, and has been used extensively for the investigation of in-stent restenosis (ISR). Due to its wide spread use, the rabbit iliac artery stent model is well characterised^8^ and the flow dynamics and vascular response compare favourably to the pig coronary artery. In particular, re-endothelialisation occurs at a rate comparable with humans in the rabbit model whereas it is accelerated in the pig.^49^

This model was used to evaluate the *in vivo* pharmacokinetics of the Xience Prime everolimus eluting stent (EEV) and the Endeavour Resolute slow-release zotarolimus eluting stent (R-ZES) in healthy arteries. The authors reported a consistent and significantly greater level of arterial drug deposition in rabbits receiving R-ZES compared to EES over a 90 day period, despite similar *in vitro* release profiles of the two stents. The difference was attributed to the increased lipophilicity of zotarolimus over everolimus, and confirms results from *ex vivo* and *in vitro* experiments. Furthermore, as in the pig studies described earlier, although the tissue drug content decreased over time, drug was still present after 90 days despite the majority having been eluted after 28 days.^74^

It is clear that the strength of *in vivo* models is that they are most relevant to the clinical situation. However, ethical concerns and cost implications mean that evaluation of drug release at the multiple time points necessary to fully characterise drug release, is necessarily limited to a small number of studies. Moreover, the majority of the models being used currently do not have disease that is comparable to the atherosclerotic plaques found in patients with coronary heart disease.

### *Ex Vivo* Models

The use of *ex vivo* models is particularly appealing, because they enable the mechanisms governing drug transport within stented arteries to be explored in a way that is simply not possible *in vivo*. This approach allows for the control of some variables, such as flow rate and pulsatility, which can be altered to closely replicate the *in vivo* situation.

**Figure 2:**
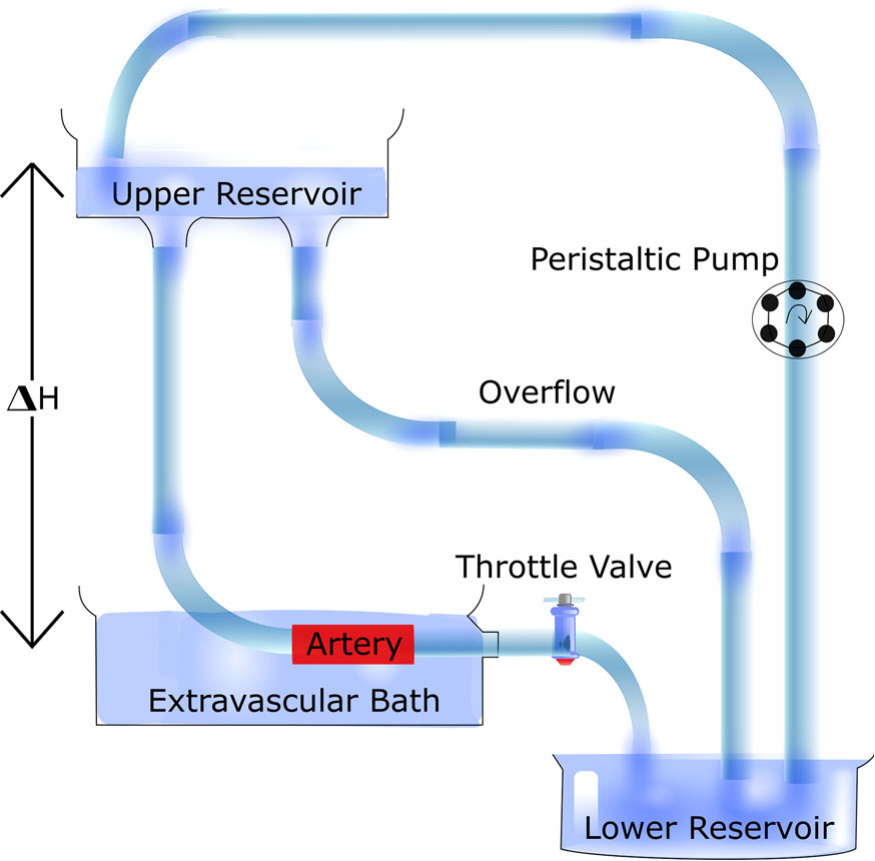
Diagram of an *ex vivo* perfusion circuit. The setup consists of an extravascular bath containing the excised artery. The bath is supplied buffer by an upper reservoir* via* tubing, into which flow is recirculated* via* a peristaltic pump. The upper reservoir height, Δ*H*, can be adjusted to alter flow rate and transmural pressure. Figure adapted from Lovich *et al*. 1995.^33^

The role of convective and diffusive transport and physical properties of drug and tissue on uptake and distribution were described in work by Creel *et al*.^9^ in *ex vivo* perfused calf carotid arteries. The setup consists of an extravascular bath into which an upper reservoir, at sufficient height to produce a pressure gradient, flows *via* tubing (Fig. 2 as described by Lovich *et al*.)^33^ In this system, it was found that the distribution of the hydrophobic drug paclitaxel was dependent on site of delivery, perivascular vs. endovascular, and time. Furthermore, paclitaxel was retained within the tissue at around 20 times higher concentration than the hydrophilic drug heparin, highlighting the importance of drug physicochemical properties in tissue uptake and distribution.^9^ Further work with this model incorporated a balloon expanded DES and highlighted the heterogeneity of drug distribution. Indeed, there were vast differences in drug concentration between segments proximal and distal to the stent struts.^23^ The importance of vessel wall multi-layered geometry and ultrastructure on drug diffusion was described using this model, and this highlighted the importance of anisotropic diffusion.^22^ The presence of disease will significantly disrupt the normal vessel structure. How this might affect drug transport is discussed later.

Drug binding and distribution have also been evaluated in excised arterial tissue incubated in drug solutions. The importance of binding sites was evident for paclitaxel which was distributed in a non-uniform fashion after 60 h incubation, whereas sirolimus was evenly bound across the tissue. These differences were determined to be dependent on the abundance and location of specific and non-specific binding sites.^32^

Whilst these models have provided useful insights into the mechanisms governing arterial drug transport and distribution, they do not mimic all aspects of the *in vivo* situation and in particular it is generally only practical to maintain such *ex vivo* set ups for up to a few days. Given these various limitations, mathematical and computational analysis of drug release have emerged as useful tools for understanding the performance of existing devices and optimising future devices.

### Computational Models

Mathematical and computational models of drug release and distribution range in complexity, from well-defined 1D simplifications that can be solved analytically, through to 2D and 3D representations that require numerical methods and finite element analysis approaches. State of the art in this field was recently reviewed by McGinty (2014).^35^ The models can be roughly separated into three categories: those which focus solely on modelling drug release in a controlled environment; those which concentrate on modelling drug transport in arterial tissue; and those which couple the drug release and tissue transport to simulate the *in vivo* environment. In the first of these categories, simple 1D mathematical models of drug release from non-absorbable polymer-coated stents have been shown to agree well with controlled experiments.^20^^,^^37^^,^^75^ In addition, 1D models have been presented for drug release from biodegradable polymer coatings^50^ and most recently from polymer free stents.^38^ In the second category, models of drug transport through arterial tissue have typically included diffusion, convection and binding phenomena.^23^^,^^62^ Finally, the third category includes models which couple drug release with tissue distribution and these show the most promise in terms of simulating *in vivo* experimental results. Various levels of complexity have been employed with the extension to 2D^6^^,^^10^^,^^76^ and 3D models,^41^^,^^65^^,^^71^^,^^78^ the inclusion of anisotropic diffusion properties,^76^ and the development of sophisticated nonlinear saturable binding models.^64^ Whilst simplified 1D models can provide useful insights, ultimately 3D coupled models which capture the full complex geometry of the stent and the arterial wall are likely to be required. The existing 3D models all make certain simplifying assumptions, such as idealising the stent geometry, neglecting convection, diffusion or binding, in considering only single or bi-layer arterial walls, or neglecting underlying disease. Nevertheless, mathematical and computational modelling have emerged as useful tools for gaining important insights into the drug release and tissue distribution processes, informing device design, and in helping ascertain the numerous parameters of the increasingly complex models. As a result, mathematical and computational modelling will likely go hand-in-hand with a combination of *in vitro*, *ex vivo* and *in vivo* experiments in advancing the design of the stents of the future.

## Modelling Impact of Atherosclerosis on Drug Release and Distribution

The majority of models described above fail to adequately capture the effects of disease, specifically the presence of atherosclerotic plaque, on drug release and distribution. This may be an important limitation, given that emerging evidence suggests that drug levels achieved within the artery wall following stenting are influenced by variations in plaque structure, composition and receptor distributions.^63^ Recent advances in intravascular imaging reveal that plaque structure and characteristics can be quite variable from patient to patient,^25^^,^^26^^,^^60^ making it likely that drug levels achieved within the artery wall will vary from patient to patient. The extent to which this variability impacts on clinical outcomes remains to be confirmed. To understand this more fully, there is thus a need to incorporate variations in plaque structure and composition into the existing models used in the investigation of stent-based drug delivery. We will therefore now go on to review the current methods available for investigating the impact of disease severity on coronary stent drug release and distribution.

### In Vitro

In recent years, a variety of approaches to incorporate the effects of disease into *in vitro* models of stent drug release have been developed. Such approaches have largely involved the modification of previously established *in vitro* models. The vessel simulating flow through cell model first described by Neubert *et al.*^46^ was adapted to assess the utility of this system to incorporate hydrophobic regions within the hydrogel. Hydrogels were modified using oil droplets or hydrophobic microparticles, to produce a material more relevant to the *in vivo*/disease situation.^58^ This initial study measured the partition coefficient, the ratio of drug content within the gel compartment and drug within the surrounding solution, of two model drugs, hydrophilic fluorescein and hydrophobic triamterene, in the hydrogels incubated in drug solutions. This modification had no effect on the partition coefficient of fluorescein; however the partition coefficient of triamterene was sensitive to the gel modification and increased 3- to 5-fold dependent on the modifying agent. Despite this there was no change in release rate or uptake of either molecule when these gels were incorporated into the vessel simulating flow cell. This was likely due to the stent polymer coating which controlled the release. Indeed, further examination of the data using finite element analysis was in agreement with the distribution pattern of triamterene when a fast release from the stent coating was assumed.^58^

The artificial vessels used thus far have been generally composed of alginate. However, recent attempts have been made to better mimic the properties of the sclerotic vessel wall by using gelatin to mimic collagen and physiologically relevant lipids to emulate lipid laden plaques in phantom vessels.^18^ In these experiments, phantom vessels were housed in syringes and the surface exposed to solutions containing hydrophilic tetracycline, or hydrophobic fluvastatin, for a period of three or four days respectively. Some aspects of drug transport were found to be dependent upon the composition of the phantom vessels, with the nature of such dependency being dependent on the physical properties of the drugs investigated. For example, the diffusion coefficient of the hydrophilic tetracycline was dependent on the gelatin concentration, and decreased as gelatin concentration increased, although was largely independent of lipid concentrations. The future use of such models should therefore ensure careful selection of the collagen level of the artificial plaque so that it closely matches that found within diseased human vessels. In contrast to tetracycline, fluvastatin transport was strongly dependent on lipid concentration, displaying decreased diffusivity at elevated lipid concentrations. The precise effect of lipid concentrations on drug transport therefore appears to be dependent on the physicochemical properties of the drug. In the same study by Guo *et al*.^18^ the fluvastatin partition coefficient increased with increasing lipid concentrations within the gel. This finding is in contrast to those reported by Tzafriri *et al*. (2010)^63^ who found an inverse relationship between lipid content and the partition coefficients of paclitaxel, everolimus and sirolimus in human (paclitaxel only) and rabbit atherosclerotic aortas. The observed differences between these studies may be due to physicochemical differences in the drugs under investigation in each study, although it is also likely to be a reflection of the increased complexity of the *ex vivo* tissues^63^ over the phantom vessels,^18^ where binding of drug to specific sites was neglected. Therefore, although important information on drug transport can be found from current artery mimics, the lack of cell specific binding within such models remains a key limitation. However, their ability to study the effects of particular components of the plaque in a controlled and systematic manner, mean that they will be useful for providing mechanistic insights into the how the transport of different drug types are likely to be differentially affected within atherosclerotic vessels.^18^

### *In Vivo*

#### Pig

There are a number of pig models available which develop atherosclerosis in response to a high fat diet. Lesions develop around 6 months, mainly in the abdominal aorta and coronary arteries.^16^^,^^66^ Furthermore, a line of pigs, with familial hyperlipidaemia syndrome (FHS) have been bred that develop atherosclerosis spontaneously. These pigs develop coronary lesions within 1 year which are characterised by fatty deposits and inflammatory and SMC infiltrates, and they progress to more complex lesions throughout the animals’ lifetime.^40^^,^^51^ Although both models have been used to evaluate efficacy, with a potential dose effect indicated in one such study,^61^ they have not so far been used for detailed investigations of drug release and distribution following stenting. However, the FHS model, combined with balloon catheter induced endothelial denudation, was used in the evaluation of coated balloon drug delivery to femoral arteries.^17^ Zotarolimus was detected within 5 min of drug delivery in the superficial femoral artery. The amount of drug had reduced by 24 h though it was still detectable after 28 days.

#### Rabbit

The New Zealand White rabbit develops atherosclerotic plaques when fed an atherogenic high fat diet either alone or combined with balloon injury.^68^ Lesions develop throughout the aorta and in the coronary and iliac arteries by around 12 weeks. The lesion characteristics and distribution are variable but are generally restricted to fibrofoamy and fibrous plaques.^44^ This model may therefore be useful for the consideration of the contribution of these plaque types to drug release and tissue distribution *in vivo*. Initial studies in the atherosclerotic rabbit iliac artery confirmed findings in humans regarding ISR, where three different DES significantly reduced neointima thickness and cellular proliferation compared to bare metal stents (BMS). Additionally, re-endothelialisation was impaired with DES compared to BMS. These outcomes compare favourably to the situation in human disease, whereas previous studies using healthy arteries from rabbits failed to detect this difference.^44^ The rabbit model of atherosclerosis has yet to be used for the evaluation of drug uptake and distribution. Nonetheless, a number of studies have assessed DES efficacy in this model and have shown important similarities to many aspects of the human response to stenting.^21^^,^^74^

### Ex Vivo

**Figure 3:**
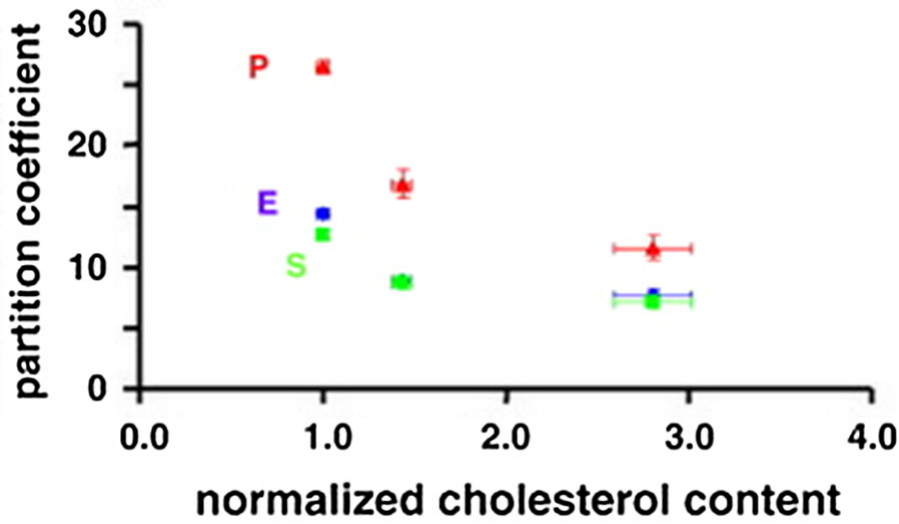
Partition coefficients of everolimus (blue), paclitaxel (red) and sirolimus (green) in atherosclerotic human aorta. Partition coefficient was greatest in the medial layer (left) where lipid content was lowest, whilst it was lowest in the intimal layer (right) where lipid content was most abundant. These data were obtained from a single sample dissected into three tunic layers, cut into 12 sections, and immersed in drug solution for 96 h (*n* = 3 for each layer and drug). Figure adapted from Tzafriri *et al*. 2010.^63^

Examination of tissue *ex vivo* has been shown to be particularly useful in determining the impact of tissue components and physiological transport mechanisms on drug release and distribution following stenting. Building on the models described earlier,^9^^,^^23^ Tzafriri *et al*.^63^ used a variety of excised vessels containing plaque to investigate the extent to which various components of disease, including alterations in tissue composition and drug-specific receptor distributions, impact upon drug transport. Samples of human aorta were obtained post-mortem from four donors and all had some degree of necrotic and calcific regions within plaques, which also contained moderate to high levels of lipid. Partition coefficients for sirolimus, paclitaxel and everolimus were evaluated in a lightly calcified sample, which was separated into its tunic layers and immersed in solutions of the drugs for 96 h. Within all three layers drug deposition was dependent on lipid content (see Fig. 3). To further validate these findings the authors calculated the partition coefficient of the medial layer of healthy calf carotid arteries which had lower lipid content than the human samples, and subsequently the partition coefficient was higher. Overall these findings were contrary to the previously held expectation that hydrophobic drugs would be better retained in a lipid rich environment.^15^^,^^37^

**Figure 4:**
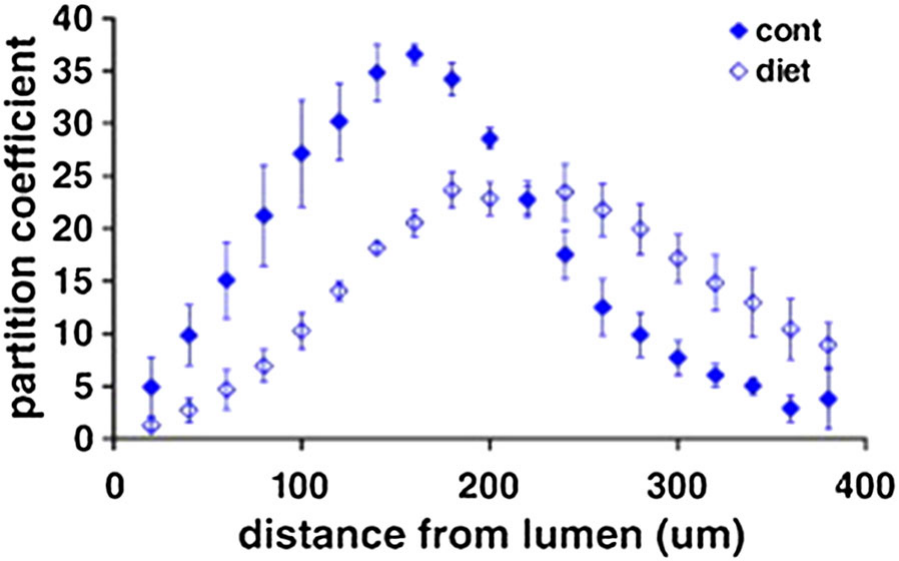
Distribution of paclitaxel across the vessel wall in an atherosclerotic rabbit aorta. Arteries incubated in paclitaxel displayed an altered distribution pattern compared to control tissues. The partition coefficient was determined in 20 *µ*m thick tissue sections spanning the intimal to adventitial side of the vessel (*n* = 3). Figure adapted from Tzafriri *et al*. 2010.^63^

In the same study,^63^ to further investigate the drug distribution pattern seen in the human tissue, a rabbit model of atherosclerosis, induced through varying degrees of dietary cholesterol and balloon injury, was utilised to produce lesions characterised by predominantly lipid lesions or more advanced sclerotic lesions. Net partition coefficient was unaltered in the injured arteries compared to control arteries for all drugs, with both drug loading time and equilibrium reached at similar points. However, fluorescently labeled paclitaxel accumulated in regions of lower lipid content in diseased arteries, similar to the human tissue. Furthermore, paclitaxel distribution was altered in the injured rabbit model when compared to controls (Fig. 4). Staining of the tissue indicated that tubulin, the target of paclitaxel, was reorganised in the diseased vessels and accumulated towards the medial layer where partition coefficient was highest. Despite the relationship between lipid and partition coefficient in the human sample, there was no alteration in distribution of everolimus in the rabbit tissues. Furthermore, FKBP12 expression was unchanged by the introduction of injury indicating that drug retention was dependent on the location and availability of specific binding sites rather than the presence of lipid.^63^ Overall, this study has highlighted the important effects that specific binding sites have on drug distribution following local arterial delivery. Given that altered distribution of such binding sites appears to accompany atherosclerosis, such effects should be incorporated into future models used in DES development so that they better account for the presence of disease.

### Computational Models

Despite the progress that has been made, very few of the existing computational approaches incorporate disease states. Of the models that do, idealised geometries and plaque characteristics have been assumed as there has been no effective way of incorporating real patient specific information into such models. In one of the first models of drug distribution through atherosclerotic plaque, McGinty *et al*.^36^^,^^37^ incorporated a highly simplified model of plaque into their 1D coupled drug release and tissue distribution model. Plaque was assumed to be homogenous, with a first order reaction kinetics model adopted to account for possible drug binding. Bozsak *et al*.^6^ accounted for disease by modelling a thickened sub-endothelial space. Most recently, Ferreira *et al*.^14^ accounted for disease by including the stiffness of the arterial wall in their model, and increasing the stiffness to simulate a diseased arterial wall. An important factor that has limited the incorporation of disease into computational models is the limited availability of the required parameters, with the majority of those in the literature derived from healthy vessels. There is thus a need to measure these parameters in diseased vessels and with the use of some of the modified *in vitro* and *ex vivo* models described earlier, this is now becoming a realistic ambition. Equally, the emergence of enhanced intravascular imaging techniques may help enable the development of computational models of stent drug release with more realistic lesion geometries.^42^

### Conclusions and Future Directions

The models described in this review have played an important role in DES research and development over many years. The insights they have provided have helped establish a greater understanding of the key elements governing stent drug release and distribution, information that has been crucial to the development of existing drug release profiles and which will help inform the development of enhanced devices in future.

It is clear from current models that drug release and tissue uptake from DES is dependent on a number of factors. *In vitro* and *ex vivo* models have highlighted that the drug physicochemical properties, release medium, the drug coating and the presence of flow can all have important effects on drug release and distribution. It is also becoming clear that drug uptake and release is altered by the presence of atherosclerosis. Indeed, *in vitro* and *ex vivo* experiments have shown drug partitioning to be greatly affected by the presence of lipid, with the availability and distribution of specific and non-specific drug binding sites also critical in this respect.

However, certain limitations remain and it is clear that there are significant opportunities to advance on current approaches. Both *in vitro* and *in vivo* models fail to fully emulate the human condition^67^ and can only provide information on drug release and uptake at a limited number of time points, often at great expense and with high technical challenge. Whilst mathematical and computational models can help address some of these limitations, current approaches in this area have still generally assumed idealised vessel geometries and compositions, meaning that they do not adequately incorporate the important effects of disease that have been revealed by *ex vivo* studies. In part, this has been due to the lack of reliable model parameters obtained from diseased vessels. However, the measurement of such parameters has now begun.^63^ In addition, the use of rodent models of atherosclerosis such as the apolipoprotein E knockout (ApoE−/−) mouse provides opportunities to carry out large scale statistically powerful studies, which may help accelerate progress in this emerging research area.^12^^,^^43^^,^^53^ Indeed, many models of stenting, using miniaturised stents, have been described in the mouse with many hallmarks of the human disease.^1^^,^^11^^,^^59^ Furthermore, the Watanabe heritable hyperlipidaemic rabbit, which is a well characterised model of atherosclerosis, displays a myriad of lesion types and may prove a useful tool in advancing this research.^13^ When taken together with advances in intravascular imaging, there is thus the opportunity to better inform the development of computational models that are more closely aligned to the disease state. Ultimately, such approaches should increasingly be used to help further our understanding of the impact of disease on stent-based drug release and distribution, thus helping in the development of more effective DES in future.

## References

[1] Ali ZA (2007). Increased in-stent stenosis in ApoE knockout mice: insights from a novel mouse model of balloon angioplasty and stenting. ArteriosclerThrombVascBiol.

[2] Baber U, Kini AS, Sharma SK (2010). Stenting of complex lesions: an overview. Nat Rev Cardiol.

[3] Bernelli C, Chan J, Chieffo A (2014). Drug-eluting stent outcomes in diabetes. Expert Rev CardiovascTher.

[4] Bierer BE (1990). 2 Distinct signal transmission pathways in lymphocytes-t are inhibited by complexes formed between an immunophilin and either Fk506 or rapamycin. Proc Natl Acad Sci USA.

[5] Biggs KB, Balss KM, Maryanoff CA (2012). Pore networks and polymer rearrangement on a drug-eluting stent as revealed by correlated confocal Raman and atomic force microscopy. Langmuir.

[6] Bozsak F, Chomaz J-M, Barakat AI (2014). Modeling the transport of drugs eluted from stents: physical phenomena driving drug distribution in the arterial wall. Biomech Model Mechanobiol.

[7] Carlyle WC (2012). Enhanced drug delivery capabilities from stents coated with absorbable polymer and crystalline drug. J Control Release.

[8] Coats P (2008). Inhibition of non-Ras protein farnesylation reduces in-stent restenosis. Atherosclerosis.

[9] Creel CJ, Lovich MA, Edelman ER (2000). Arterial paclitaxel distribution and deposition. Circ Res.

[10] Denny WJ, Walsh MT (2014). Numerical modelling of mass transport in an arterial wall with anisotropic transport properties. J Biomech.

[11] Douglas G (2013). Endothelial cell repopulation after stenting determines in-stent neointima formation: effects of bare-metal vs. drug-eluting stents and genetic endothelial cell modification. Eur Heart J.

[12] Ewart MA (2014). Altered vascular smooth muscle function in the ApoE knockout mouse during the progression of atherosclerosis. Atherosclerosis.

[13] Fan JL (2015). Rabbit models for the study of human atherosclerosis: from pathophysiological mechanisms to translational medicine. Pharmacol Ther.

[14] Ferreira, J. A.* et al*. A coupled non-fickian model of cardiovascular drug delivery system, Pre-Publicacoes do Departmento de Matematica, Universidade de Coimbra, Preprint Number 14-13

[15] Finn AV (2007). Vascular responses to drug eluting stents - Importance of delayed healing. Arterioscler Thromb Vasc Biol.

[16] Getz GS, Reardon CA (2012). Animal models of atherosclerosis. Arterioscler Thromb Vasc Biol.

[17] Granada JF (2011). Vascular response to zotarolimus-coated balloons in injured superficial femoral arteries of the familial hypercholesterolemic swine. Circ Cardiovasc Interv.

[18] Guo J (2013). Impact of artificial plaque composition on drug transport. J Pharm Sci.

[19] Hara H (2006). Role of stent design and coatings on restenosis and thrombosis. Adv Drug Deliv Rev.

[20] Hossainy S, Prabhu S (2008). A mathematical model for predicting drug release from a biodurable drug-eluting stent coating. J Biomed Mater Res Part A.

[21] Hoymans VY (2014). Long-term vascular responses to Resolute(R) and Xience V(R) polymer-based drug-eluting stents in a rabbit model of atherosclerosis. J IntervCardiol.

[22] Hwang CW, Edelman ER (2002). Arterial ultrastructure influences transport of locally delivered drugs. Circ Res.

[23] Hwang CW, Wu D, Edelman ER (2001). Physiological transport forces govern drug distribution for stent-based delivery. Circulation.

[24] Inoue T (2011). Vascular inflammation and repair: implications for re-endothelialization, restenosis, and stent thrombosis. JACC CardiovascInterv.

[25] Iqbal SN (2014). Characteristics of plaque disruption by intravascular ultrasound in women presenting with myocardial infarction without obstructive coronary artery disease. Am Heart J.

[26] Jia H (2013). In vivo diagnosis of plaque erosion and calcified nodule in patients with acute coronary syndrome by intravascular optical coherence tomography. J Am CollCardiol.

[27] Kamath KR, Barry JJ, Miller KM (2006). The Taxus drug-eluting stent: a new paradigm in controlled drug delivery. Adv Drug Deliv Rev.

[28] Kamberi M (2009). A novel accelerated in vitro release method for biodegradable coating of drug eluting stents: insight to the drug release mechanisms. Eur J Pharm Sci.

[29] Khan W, Farah S, Domb AJ (2012). Drug eluting stents: developments and current status. J Control Release.

[30] Khan W (2013). Carrier free rapamycin loaded drug eluting stent: in vitro and in vivo evaluation. J Control Release.

[31] Kuh HJ (2000). Computational model of intracellular pharmacokinetics of paclitaxel. J Pharmacol Exp Ther.

[32] Levin AD (2004). Specific binding to intracellular proteins determines arterial transport properties for rapamycin and paclitaxel. Proc Natl Acad Sci USA.

[33] Lovich MA, Edelman ER (1995). Mechanisms of transmural heparin transport in the rat abdominal aorta after local vascular delivery. Circ Res.

[34] Mani G (2007). Coronary stents: a materials perspective. Biomaterials.

[35] McGinty S (2014). A decade of modelling drug release from arterial stents. Math Biosci.

[36] McGinty S (2011). Modelling drug-eluting stents. Math Med Biol.

[37] McGinty S (2013). Modeling arterial wall drug concentrations following the insertion of a drug-eluting stent. SIAM J Appl Math.

[38] McGinty S (2015). Some design considerations for polymer-free drug-eluting stents: a mathematical approach. Actabiomaterialia.

[39] Menown IB (2010). The platinum chromium element stent platform: from alloy, to design, to clinical practice. Adv Ther.

[40] Milewski K (2012). Evaluation of efficacy and dose response of different paclitaxel-coated balloon formulations in a novel swine model of iliofemoral in-stent restenosis. JACC Cardiovasc Interv.

[41] Mongrain R (2007). Effects of diffusion coefficients and struts apposition using numerical simulations for drug eluting coronary stents. J Biomech Eng Trans ASME.

[42] Morlacchi S (2013). Patient-specific simulations of stenting procedures in coronary bifurcations: two clinical cases. Med EngPhys.

[43] Nakashima Y (1994). ApoE-deficient mice develop lesions of all phases of atherosclerosis throughout the arterial tree. Arterioscler Thromb.

[44] Nakazawa G (2011). Evaluation of polymer-based comparator drug-eluting stents using a rabbit model of iliac artery atherosclerosis. Circ Cardiovasc Interv.

[45] Naseerali CP, Hari PR, Sreenivasan K (2010). The release kinetics of drug eluting stents containing sirolimus as coated drug: role of release media. J Chromatogr B Analyt Technol Biomed Life Sci.

[46] Neubert A (2008). Development of a vessel-simulating flow-through cell method for the in vitro evaluation of release and distribution from drug-eluting stents. J Control Release.

[47] O’Brien CC (2012). Analysis of drug distribution from a simulated drug-eluting stent strut using an in vitro framework. Ann Biomed Eng.

[48] O’Brien CC (2013). Impact of flow pulsatility on arterial drug distribution in stent-based therapy. J Control Release.

[49] Perkins LE (2010). Preclinical models of restenosis and their application in the evaluation of drug-eluting stent systems. Vet Pathol.

[50] Prabhu S, Hossainy S (2007). Modeling of degradation and drug release from a biodegradable stent coating. J Biomed Mater Res Part A.

[51] Prescott MF (1991). Development of complex atherosclerotic lesions in pigs with inherited hyper-LDL cholesterolemia bearing mutant alleles for apolipoprotein B. Am J Pathol.

[52] Ragkousis GE, Curzen N, Bressloff NW (2014). Simulation of longitudinal stent deformation in a patient-specific coronary artery. Med EngPhys.

[53] Rattazzi M (2005). Calcification of advanced atherosclerotic lesions in the innominate arteries of ApoE-deficient mice: potential role of chondrocyte-like cells. Arterioscler Thromb Vasc Biol.

[54] Schiavone A, Zhao LG, Abdel-Wahab AA (2014). Effects of material, coating, design and plaque composition on stent deployment inside a stenotic artery—finite element simulation. Mater Sci Eng C Mater Biol Appl.

[55] Schwartz RS (2002). Drug-eluting stents in preclinical studies: recommended evaluation from a consensus group. Circulation.

[56] Schwartz RS (2008). Drug-eluting stents in preclinical studies: updated consensus recommendations for preclinical evaluation. Circ Cardiovasc Interv.

[57] Seidlitz A (2011). Examination of drug release and distribution from drug-eluting stents with a vessel-simulating flow-through cell. Eur J Pharm Biopharm.

[58] Semmling B (2013). Development of hydrophobized alginate hydrogels for the vessel-simulating flow-through cell and their usage for biorelevant drug-eluting stent testing. AAPS PharmSciTech.

[59] Simsekyilmaz S (2013). A murine model of stent implantation in the carotid artery for the study of restenosis. J Vis Exp.

[60] Tan A (2014). OSA and coronary plaque characteristics. Chest.

[61] Tellez A (2014). Peri-strut low-intensity areas in optical coherence tomography correlate with peri-strut inflammation and neointimal proliferation: an in vivo correlation study in the familial hypercholesterolemic coronary swine model of in-stent restenosis. Coron Artery Dis.

[62] Tzafriri AR, Levin AD, Edelman ER (2009). Diffusion-limited binding explains binary dose response for local arterial and tumour drug delivery. Cell Prolif.

[63] Tzafriri AR (2010). Lesion complexity determines arterial drug distribution after local drug delivery. J Control Release.

[64] Tzafriri AR (2012). Stent elution rate determines drug deposition and receptor-mediated effects. J Control Release.

[65] Vairo G (2010). Drug release from coronary eluting stents: a multidomain approach. J Biomech.

[66] Vilahur, G., T. Padro, and L. Badimon, Atherosclerosis and thrombosis: insights from large animal models. J Biomed Biotechnol, 2011. 10.1155/2011/907575 PMC3022266 21274431

[67] Virmani R (2000). Lessons from sudden coronary death: a comprehensive morphological classification scheme for atherosclerotic lesions. Arterioscler Thromb Vasc Biol.

[68] Wang Y (2011). Gene delivery of soluble vascular endothelial growth factor receptor-1 (sFlt-1) inhibits intra-plaque angiogenesis and suppresses development of atherosclerotic plaque. ClinExp Med.

[69] Watanabe T (2014). Integrity of stent polymer layer after drug-eluting stent implantation: in vivo comparison of sirolimus-, paclitaxel-, zotarolimus- and everolimus-eluting stents. Cardiovasc Interv Ther.

[70] Watt J (2013). Succinobucol-eluting stents increase neointimal thickening and peri-strut inflammation in a porcine coronary model. Catheter Cardiovasc Interv.

[71] Weiler JM, Sparrow EM, Ramazani R (2012). Mass transfer by advection and diffusion from a drug-eluting stent. Int J Heat Mass Transf.

[72] Wiebe J, Nef HM, Hamm CW (2014). Current status of bioresorbable scaffolds in the treatment of coronary artery disease. J Am Coll Cardiol.

[73] Yang C, Burt HM (2006). Drug-eluting stents: factors governing local pharmacokinetics. Adv Drug Deliv Rev.

[74] Yazdani SK (2013). Preclinical evaluation of second-generation everolimus- and zotarolimus-eluting coronary stents. J Invasive Cardiol.

[75] Zhao HQ (2012). A theoretical model to characterize the drug release behavior of drug-eluting stents with durable polymer matrix coating. J Biomed Mater Res Part A.

[76] Zhu X, Braatz RD (2014). Modeling and analysis of drug-eluting stents with biodegradable PLGA coating: consequences on intravascular drug delivery. J Biomech Eng.

[77] Zhu W (2009). In-vitro release of rapamycin from a thermosensitive polymer for the inhibition of vascular smooth muscle cell proliferation. J Bioequiv Available.

[78] Zunino P (2009). Numerical simulation of drug eluting coronary stents: mechanics, fluid dynamics and drug release. Comput Methods Appl Mech Eng.

